# “Our Generation Is Trying to Break Some of That Resistance to Emotions”—A Mixed-Methods Pilot Examination of Tuning in to Kids for Black Parents of Preschoolers in the United States

**DOI:** 10.3390/children11070803

**Published:** 2024-06-30

**Authors:** Briana J. Williams, John S. Carlson

**Affiliations:** College of Education, Michigan State University, East Lansing, MI 48823, USA; will3460@msu.edu

**Keywords:** parenting program, Black parents, early childhood, mixed methods, pilot study

## Abstract

Background: A growing body of literature examines the utility of emotion-focused parenting programs, as behaviorally based programs currently dominate the parenting literature. Few of those studies examine differences in how Black parents may benefit. This mixed-methods pilot study examined preliminary fidelity, efficacy, and acceptability of Tuning in to Kids (TIK), an emotion-focused parenting program targeting parenting practices and children’s emotion regulation through a strengths-based approach. Methods: Pre, post, and one-month follow-up measurements were collected from 21 parents in the United States who were randomly assigned to a treatment (i.e., TIK) or waitlist control group. They were assessed across several self-report parent measures (parental emotion regulation, emotion socialization parenting practices and beliefs) and parent-report of children’s social-emotional competence. Parents in the TIK group completed interviews to further understand their experience participating in the intervention. Results: Descriptive analyses showed general improvements and positive change in parenting practices, beliefs, parental emotion regulation, and children’s self-regulation. Large effect sizes indicate reductions of parents emotion dismissing and distressed reactions to children’s negative emotions. TIK was overall rated as a highly acceptable intervention. Parent interviews offer essential information to provide context to Black parents’ experiences utilizing TIK as well as themes related to challenges in raising Black children with self-regulation difficulties. Conclusions: Overall, these preliminary mixed-methods outcomes suggest that TIK is a promising parenting program to improve Black parents’ emotion regulation, emotion coaching beliefs and positive parenting practices. Further research is needed to investigate the effectiveness of TIK and other emotion-focused parenting programs with Black parents and assess the necessity of future cultural adaptations.

## 1. Introduction

Emotion socialization is broadly defined as how parents and other caregivers teach their children to recognize, control, express, and/or suppress their emotions [1], which reflects the values of a given familial or cultural context, indicating that one model of emotion socialization does not fit all. Black parents, for example, may socialize their children differently than their non-Black peers as they also socialize their children to navigate systemic barriers, such as racism [2,3]. Research indicates that Black parents appear to use both supportive (e.g., encouraging emotional expressiveness) and “unsupportive” (e.g., suppression of negative emotions) parenting practices interchangeably as an adaptive method to protect their children from negative social perceptions and to manage negative emotions in a variety of contexts [4,5,6,7].

In the broader emotion socialization research, unsupportive parenting practices include those that seek to minimize a child’s emotions or punish them for expressing negative emotions, such as anger or fear [1]. However, for Black families, parenting practices that may appear unsupportive to White families can be protective for Black families [4]. For example, in specific settings, a Black parent may view expressions of negative emotion in public as unacceptable due to fears of being misinterpreted by peers or other members of the majority race [8,9]. Raval and Walker [10] expanded on Eisenberg and colleagues’ [1] heuristic model of parental emotion socialization to highlight the importance of culture in understanding parenting values and practices across cultural groups. The expanded framework suggests that Black parents may use varying parenting practices concerning how others view the child [10]. These parents may seek to protect their children from being mislabeled due to undeserved stereotypes of Black children (e.g., hyperactivity and high energy levels) [11]. When discussing emotion socialization in Black and African American families, one must consider the influence of culture on the value placed on emotional expression [5].

Morris and colleagues presented a Tripartite Model that introduces the familial impact of children’s emotion regulation and adjustment [12]. The model emphasizes how children’s emotion regulation is impacted by observing and modeling those in their environment, parenting practices, and parenting styles. Dunbar and colleagues proposed a conceptual model that integrates racial/ethnic and emotion socialization specifically for African American families and builds on the Tripartite Model [13]. They posit that racial/ethnic socialization and emotion socialization overlap in that Black families are often tasked with emotionally socializing their children within the context of preparing for bias to overcome racism. Findings in the emotion socialization literature identify unsupportive parenting practices as adversely impacting White children’s emotion regulation and competence [14]. However, those parenting practices do not have the same negative outcomes for Black children [4]. Specifically, suppression of their children’s negative emotions in preparation for racism and bias is associated with better outcomes for Black children, such as reduced externalizing behaviors. Regardless of preparation for racism and bias, however, the suppression of negative emotions resulted in increased the internalization of problems [15]. Wu and Hooper explored similar associations between parental emotion coaching beliefs and children’s internalizing and externalizing behaviors in a sample of Black and White parent–child dyads [16]. Their findings support that parental emotion coaching beliefs were associated with fewer internalizing tendencies for Black children with low sensitivity to emotion socialization and their environment, whereas Black children with higher sensitivity exhibited more internalizing tendencies. These associations were not found for the White children in the sample. In Black families, supportive and unsupportive parenting practices may not have the same meaning as in White families. In this sense, practices that may appear unsupportive to White families can be adaptive for Black families [4]. For example, a Black parent may view expressions of negative emotion in public as unacceptable due to fears of being misinterpreted by peers or other majority culture members [8,9]. Nelson and colleagues found that Black children who were socialized to express negative emotions were more likely to have poorer academic performance and social-emotional competence than their White peers [7]. Parents may seek to protect their children from being mislabeled due to undeserved stereotypes of Black children (e.g., hyperactivity and high energy levels) [11]. Future research investigating emotion socialization within Black samples should consider the cultural implications of the outcomes in interpreting results.

Few evidence-based interventions (EBIs) have examined parenting practices for Black families. EBIs should consider how interventions meet an individual’s personal needs and culture. However, emotion-focused interventions are conducted with predominantly White samples, suggesting the need to investigate the evidence to support using emotion-focused interventions for Black parents. While Black parents are often included in the larger samples, the results of these intervention studies are not disaggregated by race or ethnic group, limiting the ability to examine the effectiveness of EBIs for Black families.

Interventions that support Black parents focus on various issues, including promoting social competence (e.g., Incredible Years) [17], reducing conduct behaviors (e.g., Triple P) [18], and supporting racial socialization (e.g., EMBRace) [19]. A mixed-methods study of Triple P (i.e., Positive Parenting Program) examined parents’ changing attitudes to spanking after exposure to the program [20]. Qualitative results indicated that some Black parents reconsidered using spanking as discipline after completing Triple P. In the future, they would try to work through a problem with their child before resorting to spanking While the Triple P program does not directly address spanking or physical punishment, it does emphasize positive alternative strategies parents can use. These findings suggest that parenting programs are effective when teaching acceptable behaviors rather than dissuading practices perceived as negative (e.g., suppressing negative emotions and behaviors). Interventions like Triple P can effectively educate parents with alternative parenting practices to support their children’s development [20].

Two group-based parenting programs have examined social-emotional change in early childhood in Black families. The Mommy and Me Play Intervention is a strengths-based program to support preschoolers’ social-emotional competence through play with their mothers [21]. Mothers were paired in mother–child dyads (i.e., two mothers and two children) in a structured play group, where each parent was assigned to either the Helper or Helpee group. Helpers were those with higher skills in pre-intervention, and Helpees were less skilled in pre-intervention. Mothers were not aware of their assigned condition during the study. Mother-child dyads were instructed to “Help your children play together and see what you can learn from watching and playing with each other”. Following the intervention, Wright and their research team observed that mothers assigned to the Helpees group demonstrated positive changes in responsivity and warmth toward their child. Overall, Helpees (i.e., less-skilled mothers at baseline) made the most significant gains after the intervention, suggesting the benefit of a collaborative peer model [21].

Another program developed to support Black parents and their children is the Black Parenting Strengths and Strategies (BPSS) Program [22]. BPSS was adapted from the Parenting the Strong-Willed Child (PSWC) [23] an evidence-based intervention developed to improve parent–child relationships, teach parents behavior management, and improve children’s behaviors. BPSS uses culturally specific content related to racial socialization and the core of the PSWC to support Black parents. In a pilot of Black primary caregivers of 5- and 6-year-olds (*N* = 38), Coard and colleagues examined outcomes related to parenting practices, children’s psychosocial functioning, and parents’ satisfaction and perceptions of the intervention [22]. Parents were highly satisfied with the program, and 100% of participants indicated they would recommend it to a friend. Parents in the BPSS group reported significant increases in positive parenting practices and decreased harsh discipline compared to parents in the control group. Parents also reported significant decreases in their child’s problem behaviors compared to parents in the control group, who reported increases in their child’s externalizing behavior. These findings suggest that parenting programs tailored to cultural contexts successfully support minoritized parents. While important, this study did not specifically examine parenting practices related to emotion socialization, suggesting future research’s importance in examining these practices within a cultural context.

Emotion socialization theory posits that children’s emotional competence is directly related to their parent’s ability to regulate their emotions [1].Parents who struggle with emotionality may be unaware of their emotions and thus cannot always respond to their children’s negative emotions in a supportive way [24]. Research in a predominantly Black sample demonstrated that parents who struggle with emotion dysregulation are more likely to invalidate their children’s emotional expression, which is associated with increased internalizing and externalizing symptoms in adolescent children [25]. Labella explored empirical research on parental emotion socialization in Black families and discovered that the celebration and restriction of children’s emotions coincide closely with Black families, which differs from White, middle-class samples [2]. Additional intervention research on emotion socialization practices in early childhood within Black families is needed to understand how clinicians and mental health professionals can support families.

Emotions can be linked to certain parenting styles and behaviors. Gottman and colleagues developed the parental meta-emotion philosophy (PMEP), which broadly suggests that parents’ beliefs and values of emotion influence their emotion socialization practices, which are subsequently guided by their abilities to regulate and express emotions [3,26]. The PMEP provides a foundational framework for this research, integrating parents’ beliefs of emotions and their impact on children’s emotional competence and understanding. Gottman’s PMEP provides the basis for emotion coaching (EC)—a parenting philosophy used to support children’s relationships with adults and improve their social-emotional difficulties [3,27]. Gottman and colleagues outlined five components of EC that support children’s emotional intelligence: (1) Being aware of children’s emotions, (2) recognizing emotion as an opportunity for intimacy and teaching, (3) listening emphatically and validating the child’s feelings, (4) helping the child label the emotions, and (5) setting limits while helping the child problem-solve [28]. Black parents consistently support overtly expressing positive and negative emotions [5,29] suggesting a potential strength in being aware of children’s emotions and helping them verbally label emotions. Overall, Black parents who employ an EC style report reduced internalizing symptoms in their children between the ages of seven and nine [30]. Crandall and colleagues found a negative correlation between African American mothers’ EC and their children’s anxiety symptoms, differing from European American mothers whose self-reported EC was positively correlated with their children’s anxiety symptoms [31].

In recent years, research has begun to focus on the positive effects of EC styles on young children’s emotional development with great success in predominantly White samples [32,33]. Evidence suggests that the effects of EC support the idea that preschoolers in high-risk environments benefit more from EC than peers in environments of less risk [33]. Integrating the PMEP framework with EC strategies can influence children’s emotional awareness, expression, and regulation, profoundly impacting a child’s future developmental trajectory [26]. This framework sets the stage for parents’ impact on children’s emotion socialization; however, it does not consider additional barriers Black parents may face to develop the awareness and acceptance necessary to increase their ability to coach children through emotion dysregulation. While EC parenting programs have demonstrated effectiveness in promoting positive parenting practices [32,34] and reducing conduct behavior problems of young children [35,36] limited research has examined how these programs affect children’s emotional competence and change parents’ self-reported emotion regulation processes.

While many parenting programs have components of emotion-focused content, more research is needed on programs that directly target parental emotion socialization in early childhood through prioritizing parents’ understanding of their own emotions. Tuning in to Kids (TIK) is a suite of programs developed to support the needs of parents/caregivers from early childhood to adolescence [37]. TIK aims to improve parenting, strengthen the parent–child relationship, and improve children’s emotional competence and behavior. TIK differs from the previously mentioned emotion socialization programs because the goal is not only to change children’s emotional competence. The TIK suite aims to help parents understand their emotions and their impact on their children. This aim differentiates it from other programs because ample time is spent guiding caregivers on their own emotional experiences and emphasizes the role parents’ emotion regulation and expression play in children’s emotion socialization. TIK was developed to be delivered in a group format, with one or two certified facilitators leading groups of 8 to 12. TIK has been examined as a program to promote positive parenting practices and reduce children’s conduct behaviors [38,39] and internalizing symptoms (i.e., anxiety) [40]. TIK has reduced parent-reported ED beliefs and practices and increased EC beliefs. Few studies have examined significant improvements in children’s emotional knowledge post-intervention [41]. In addition to improving parental practices, parents have reported increased self-reported empathy compared to parents in control groups [42,43,44,45] and greater satisfaction and efficacy in parenting [34]. The flexibility of the TIK manual and its content lends itself to opportunities for cultural adaptations. In recent years, researchers in China [46,47] and Norway [48] examined the efficacy of a culturally adapted TIK for mothers of preschoolers, demonstrating that participation in the program was associated with increased parental involvement and use of EC and encouragement in response to their children’s emotions. In addition to TIK’s existing literature that supports parents’ and children’s positive outcomes, there is limited information about its effectiveness being delivered virtually. To our knowledge, only one study has examined the effects of a virtually delivered TIK. Given the limited information and research on the impact of TIK in the U.S., additional research should evaluate these programs’ fidelity, effectiveness, and acceptability with a more diverse sample [49,50].

Parenting programs support and promote parents’ understanding of children’s social–emotional development and provide them with tools to respond to children’s strong emotions. We examined preliminary outcomes of parent engagement, treatment efficacy, and acceptability of Tuning in to Kids delivered virtually using a videoconferencing platform. This pilot study supports ongoing efforts to incorporate culturally responsive adaptations in future research. Pilot studies examine research protocols and experimental methods in preparation for a large-scale main trial [51,52]. This pilot study contributes to the literature in three ways. It extends the current TIK literature by (a) examining the fidelity and acceptability of this emotion-focused intervention within a small sample of U.S.-based Black parents, (b) comparing the effects of TIK with a waitlist control group (WLC), and (c) collecting qualitative evidence of this sample of Black parents’ experiences receiving the intervention. Qualitative interviews explored Black parents’ perceptions of the program’s alignment with their parenting philosophies. We also employed a mixed methods design to extend the current emotion socialization intervention literature to a diverse population.

## 2. Materials and Methods

### 2.1. Participants

Parents were recruited through communication with community partners in early childcare settings in Michigan and through social media posting in parenting groups and personal networks. Eligible participants for this study included parents who identify as Black and have a child between the ages of 3 and 5 years old. Parents were asked to indicate their specific ethnic identities within the African diaspora. Inclusion criteria included parents who rated their child as having difficulty with self-regulation (based on the Self-Regulation subscale on the DECA-P2, a raw score of 20 or less). Participants were also eligible if they spoke English as a first language and had access to a computer with internet access to participate in the weekly sessions over videoconferencing.

The initial aim for this pilot was to recruit and enroll 20 parents and randomly assign them to either the intervention or waitlist control group. One hundred twenty-six participants were assessed for eligibility, and 103 were excluded from the study due to not meeting inclusion criteria (e.g., child too young or too old, high self-regulation rating, not identifying as Black/African American), not complete the full consent or the pre-survey (*n* = 4), being flagged as a potential bot by the Qualtrics data capture system (*n* = 6), or for completing the screening survey after the target sample size was achieved (*n* = 27). The 23 parents who agreed to participate were randomly assigned to either the treatment group (*n* = 11) or the waitlist control group (*n* = 12). Two parents dropped out of the study before beginning the intervention due to last-minute scheduling conflicts and thus did not complete post-measures. In total, 21 parents (i.e., 9 in the treatment group and 12 in the WLC group) enrolled in the study and their outcomes explored. Six of the parents in the treatment group also completed a parent interview and are included in the qualitative analyses. These parents (M = 32.2 years, SD = 5.5) completed information for a child between the ages of 3 and 5 years old (M = 43.5 months, SD = 7.8) who were rated as having low self-regulation skills. Demographic characteristics of the 21 participants who completed the study are presented in Table 1. The TIK group involved parents of older children (4 years) when compared to those randomly assigned to the WLC control group (3 years, 4 months). In addition, the WLC control group contained more biological fathers and parents with part-time employment compared to the TIK group.

### 2.2. Procedures

This study was approved by the Michigan State Institutional Review Board (Study#00007571). Recruitment was initially restricted to local efforts in clinical and daycare settings, though challenges in posting recruitment fliers and connecting with families expanded efforts to online means. An electronic flyer was shared on social media sites, including parenting groups and groups for mental health professionals working with children and their families. Eligible parents agreed to complete data collection measures at pre- and post-intervention, along with a 1-month follow-up data point one month after the intervention ends. Parents were randomly assigned to either the TIK or WLC group. Additionally, parents assigned to the treatment group agreed to complete a video-recorded parent interview following the 1-month follow-up data collection. Parents in the TIK group coordinated schedules with the TIK facilitator and were asked to attend six 2-h sessions between the pre– and post–data collection time points. Parents in both groups received the TIK program, with the WLC group receiving the intervention after the 1-month follow-up surveys. Quantitative and qualitative data were collected sequentially; however, analyses of the quantitative data were not used to inform the qualitative interviews and data collection.

### 2.3. Intervention

The TIK program is a manualized program that includes six 2-h sessions facilitated by a certified trainer in a group format. TIK facilitators attended a virtual three-half-day professional development workshop to learn and understand the foundations of Emotion Coaching and implementation of TIK. Guidance was provided to support facilitators delivering the program for in-person and virtual groups, including using breakout rooms for greater peer collaboration between caregivers and using polls and surveys to increase engagement and feedback about the program. These sessions were held virtually to reduce barriers to accessing the service (e.g., transportation issues, busy schedules) and reduce contact due to the COVID-19 global pandemic [53]. Facilitators followed a manual with core material that was followed sequentially in addition to optional materials that can be delivered at any time based on parent needs. Each session covers a different topic related to understanding emotions and how to coach children through their emotional experiences. Table 2 highlights the content of each of the six TIK sessions. Participants were provided with electronic packets of materials to follow during the facilitated sessions, which included handouts, resources, and worksheets for caregivers to use with their children outside the intervention sessions. Each two-hour session consisted of a combination of (a) psychoeducation of children’s emotional development, (b) incidental learning, (c) role-playing, (d) DVD and video materials, (e) handouts, and (f) take-home activities, which all work together to diversify the teaching of the program and accommodate to fit the needs of different parenting and learning styles [37]. Between each weekly session, parents were asked to practice implementing their new skills and reflect on the topics covered. During each session, parents were asked to indicate which home activities they completed between sessions and their experience implementing them at home. Parents in the TIK group were sent private Zoom (Zoom Video Communication, Inc., San Jose, CA, USA) links for the 6-week program delivery.

### 2.4. Measures

#### 2.4.1. Treatment Engagement and Fidelity

Participant engagement was measured by documentation of session attendance and during the parent interview. After each session, fidelity checklists were completed by one of the graduate research assistants (GRA) to ensure the facilitator’s adherence to implementation quality. Checklists allowed the facilitator to address missing or modified content for future sessions. Each checklist contains the necessary topics for the facilitator to cover in each session and activities (e.g., role-play, small group discussions). The TIK facilitator manual provides formal fidelity checklists to note which activities are completed, as indicated by “yes” or “no”. A percentage of treatment fidelity was computed for each session, and an average was calculated across all sessions (see Table 2). Full fidelity (100%) to the training model was obtained while each session involved six to nine parents. These topics integrated emotion coaching and child development to support parents’ understanding of their child’s emotional difficulties.

#### 2.4.2. Treatment Efficacy

The Difficulties in Emotion Regulation Scale (DERS) is a 36-item self-report assessing the respondent’s emotional acceptance, ability to regulate emotion, perceived ability to engage in goal-directed behaviors when distressed, and emotional awareness [54]. Respondents rate how often the item applies from 1 (almost never; 0–10%) to 5 (almost always; 91–100%). The DERS consists of six subscales: (1) nonacceptance of emotional responses, (2) impulse control difficulties, (3) difficulties engaging in goal-directed behavior, (4) lack of emotional awareness, (5) lack of emotional clarity, and (6) limited access to emotion regulation strategies. Average subscale scores were computed at pre–, post–, and 1-month follow-up to explore outcomes from this pilot study. Higher scores indicate more significant challenges in emotion regulation. For African Americans, there were no differences across gender or ethnicity, demonstrating a high overall internal consistency (α = 0.94) and strong internal consistency across each subscale with Cronbach’s alpha of 0.89, 0.82, 0.86, 0.82, 0.78, and 0.87, respectively [55].

The Parental Emotional Style Questionnaire (PESQ) is a parent-report measure examining parental beliefs about a child’s ability to cope with various emotions (e.g., fear, anger, sadness) [56]. The PESQ is a 21-item measure adapted from the Maternal Emotional Style Questionnaire [57] to broaden the items for caregivers other than mothers. The EC subscale includes 11 items (e.g., “When my child is angry, it is a time for getting close”). The ED subscale includes 10 items (e.g., “I prefer my child to be happy rather than overly emotional”). Each item is rated on a 5-point Likert scale from 1 (strongly disagree) to 5 (strongly agree). Subscale scores were calculated, with higher scores on the EC being associated with high EC beliefs and practices and high ED scores indicating high instances of ED beliefs and practices. Scores on the EC subscale were used in the formal analyses, while scores on the ED subscale were examined informally. Subscales of the PESQ have good internal consistency across time for ED (α = 0.78 to 0.84) and EC (α = 0.82 to 0.87) [44].

The Coping with Children’s Negative Emotions Scale (CCNES) measured parents’ emotion socialization practices across specific situations [58]. The CCNES is a 72-item tool assessing how parents respond to 12 scenarios across six subscales: emotion-focused reactions (EFR), problem-focused reactions (PFR), distressed reactions (DR), punitive reactions (PR), expressive encouragement (EE), and minimization reactions (MR). Parents provided ratings on a 7-point scale from 1 (very unlikely) to 7 (very likely) to indicate how likely they were to respond to their child in several ways. Twelve scenarios include responses from each subscale. Fabes and colleagues report each scale as having good test-retest reliability (α = 0.69 to 0.85), construct validity, and stability over 4 months [59].

The Devereux Early Childhood Assessment for Preschoolers, Second Edition (DECA-P2) is a 38-item strengths-based assessment measuring social-emotional competence, behavior concerns, and risk factors for young children between the ages of 2 and 5 years old [60]. Twenty-seven items comprise a Total Protective Factors (TPF) scale, divided into three subscales—Initiative, Self-Regulation (SR), and Attachment/Relationships. Raw scores on the DECA-P2 are converted into *T*-scores. TPF *T*-scores of 60 or above indicate a Strength, scores between 41 and 59 indicate Typical Functioning, and scores below 40 are Areas of Need. An 11-item scale represents parent-reported Behavior Concerns (BC), where a T-score above 60 indicates an Area of Need, and scores of 59 or below are considered Typical. Parents respond to the items using a 5-point Likert scale from 0 (never) to 4 (very frequently). Test-retest reliability is acceptable and strong across the TPF and BC scales with strong correlations between same-parent ratings of their children at different time points, *r* = 0.88, *p* < 0.01 and *r* = 0.78 and *p* < 0.01, respectively [60]. Data suggests negligible differences between Black, White, and Hispanic children.

#### 2.4.3. Treatment Acceptability

The Treatment Evaluation Questionnaire-Parent (TEQ-P) is a 21-item parent-rated acceptability measure [61]. Parents in the treatment group completed the TEQ-P post-intervention to assess their perceived acceptability of TIK. The three subscales of treatment are Acceptability (11 items), Effectiveness (8 items), and Time Required to complete the intervention (2 items). Parents provided ratings of their experience in the intervention on a 6-point Likert scale from 1 (strongly disagree) to 6 (strongly agree). Total scores can range from 21 to 126, with 110 or higher indicating high parent-reported acceptability [62]. Scores indicate adequate ratings for each subscale at or above 55, 36, and 9 for acceptability, effectiveness, and time required, respectively. The TEQ-P was adapted from the Treatment Evaluation Inventory (TEI) [63], which has been shown to have high internal consistency (α = 0.97) and construct validity [8].

### 2.5. Qualitative Interviews

Parent interviews were conducted post-intervention with parents in the treatment group to assess their perceived efficacy, barriers, treatment acceptability, and perspectives on TIK’s alignment with their culture and parenting philosophies. Interview questions regarding effectiveness, barriers, and acceptability were developed to align with subscales from the TEQ-P and previous researcher questions to gather information related to the acceptability and feasibility of the intervention [9]. Additionally, parents were asked about their thoughts on the intervention’s efficacy, delivery, and alignment with their personal and cultural views of parenting. The data collected from parent interviews provide additional information from the parents’ perspectives that may not be apparent from the quantitative data. Following the last intervention session, the GRA conducted the interviews via Zoom after collecting.

### 2.6. Data Analysis

#### 2.6.1. Quantitative Data

Quantitative data were analyzed using descriptive statistics to measure treatment facilitator fidelity (i.e., percentages of items completed from the fidelity checklists), averages of attendance at the six sessions for the TIK group, and average scores of treatment acceptability. Outcome measures were analyzed using descriptive statistics (i.e., means and standard divisions) and effect size estimates given the small sample sizes, which prevented the use of parametric statistical techniques. Conducting analyses to provide effect sizes is a useful metric to document and quantify meaningful outcomes for pilot studies with small sample sizes [50].

#### 2.6.2. Qualitative Data

The interview audio recordings were initially transcribed using the Otter.ai Pro software (Otter.ai, Inc, Mountain View, CA, USA) and were checked for accuracy by a GRA. The transcripts were analyzed using a reflexive inductive thematic analysis (ITA) approach [64]. The reflexive approach allows the data to drive the themes and codes from the interview rather than use predetermined codes to match the interview content [65]. The ITA approach allowed the data to speak for itself and provide additional context to the quantitative outcomes. Reflexive approaches diverge from traditional coding analyses as researchers’ subjectivity is essential as a source of analysis rather than a “bias” that should be controlled and measured. The first author led the research team in a six-phase process to examine the patterns in the qualitative data and guide the analysis [65]. The first phase of the analysis consisted of the PI and GRAs becoming familiar with the data by listening to the audio recordings and reading the transcripts. The next two phases allowed the research team to generate initial codes emerging from the data (e.g., “difficulty using emotion coaching”) and use those codes to begin capturing broad themes from the codes (e.g., “changes in child behavior”). Through an iterative process, the team continued to review the potential themes and their relationship to emergent codes. In this phase, the first author examined whether certain codes should be themes and vice versa. The fifth phase consisted of naming and defining the themes to ensure they were distinct or combined if necessary. The final phase is producing the data report, including extracting example quotes from participants.

## 3. Results

### 3.1. Quantitative Results

All acceptability and outcome data from the TIK and WLC groups are captured in Table 3.

#### 3.1.1. Treatment Fidelity and Attendance

Seven of the nine parents (78%) who received the TIK program completed the program as determined by attending five or more sessions [43]. Four parents attended all six sessions, three attended five sessions, one attended four, and one parent attended only two. The TIK facilitator adhered to the program manual with strong fidelity at 100% across all sessions, meeting the criteria of adequate adherence greater than 80% [66]. Some sessions included optional activities, making the per-session fidelity percentages exceed 100% in some cases.

#### 3.1.2. Efficacy

A comparison of changes in means and standard deviations across time and by group showed anticipated improvement for all subscales of the DERS for parents in the TIK group. The effect size for goal-directed behavior ($\eta_{p}^{2}=0.14)$ was large, and nonacceptance of emotional responses ($\eta_{p}^{2}=0.10)$, lack of emotional awareness ($\eta_{p}^{2}=0.08)$, and limited access to emotion regulation strategies ($\eta_{p}^{2}=0.09)$ demonstrated medium effect sizes. Impulse control difficulties ($\eta_{p}^{2}=0.03)$ and lack of emotional clarity $\left(\eta_{p}^{2}=0.05\right)$ both demonstrated small effect sizes. Overall, improvement in overall difficulties in emotion regulation demonstrated a medium effect size ($\eta_{p}^{2}=0.08).$

Parent-reported changes in emotion coaching, emotion dismissing, and positive parenting practices were more variable. Large effect sizes were seen in improvements in emotion dismissing $\left(\eta_{p}^{2}=0.20\right)$ and emotion-focused reactions $\left(\eta_{p}^{2}=0.17\right)$. The remaining subscales demonstrated medium effect sizes, except for emotion coaching $\left(\eta_{p}^{2}=0.01\right)$, which was a small effect size. Parent-reported child outcomes on the DECA-P2 demonstrated a large effect size in increased self-regulation $\left(\eta_{p}^{2}=0.21\right)$, with the rest showing medium effect sizes.

#### 3.1.3. Treatment Acceptability

The average total score from the TEQ-P (M = 95.7, SD = 7.3) indicates overall moderate acceptability yet did not meet the threshold for high acceptability (i.e., 110) for the TIK group. One parent-rated their acceptability as meeting the threshold for high acceptability, with a score of 111. Six parents’ acceptability ratings were slightly under the threshold (score ranges between 94 and 99), indicating moderate acceptability. Two parents’ acceptability ratings were considered adequate overall acceptability (84 and 89). Subscales on the TEQ-P of Acceptability (M = 52.3, SD = 4.5), Effectiveness (M = 34.5, SD = 4.5), and Time Required (M = 8.8, SD = 1.0) were compared to satisfactory average ratings of 55, 36, or 9, respectively. Overall, most parents found Tuning in to Kids an acceptable and feasible program.

### 3.2. Qualitative Results

Six parents from the TIK group participated in parent interviews. Of the remaining three parents, one canceled their interview and did not wish to reschedule, one did not respond to multiple attempts to schedule an interview, and the last parent did not attend four scheduled interviews, resulting in them not completing the interview. Analysis of the parent interviews yielded seven themes related broadly to intervention engagement and barriers, effectiveness (i.e., parenting practices and emotion regulation, child behavior and regulation, parent–child relationship), acceptability (i.e., peer support), and perceptions of cultural and values-alignment of TIK for Black parents of preschoolers. Themes and corresponding codes are presented in Table 4. To maintain the integrity of the parents’ responses and qualitative interviews, direct quotes were edited only for clarity (i.e., removing repeat words) and were cut for length.

#### 3.2.1. Theme 1: Intervention Engagement and Barriers

Allowing facilitated discussions between parents in the program was noted as a key component of parents’ engagement during the virtually delivered TIK program. For example, one parent shared how important it was to discuss with the other parents outside of the lecture-style content, stating, “I really liked more when we were bouncing ideas off of each other as parents more so than like the lecture style”. They appreciated the opportunity to have questions posed by the lead facilitator, which was flexible for “pulling from us as participants I felt when she was discussing new concepts or a strategy to approach and … there was definitely freedom I felt, if we did have a question about something”. Openness to participate flexibly facilitated discussions with parents in the group through multiple modes of communication (i.e., unmuting, typing in the chat, using Jamboard [Google, Menlo Park, CA, USA]). On the other hand, when discussing the virtual nature of the program, this same parent noted that variability in other participants’ engagement impacted how they interacted with the material, asking, “How you could tell when people were really engaged, and certain people were engaged versus other parents where maybe they weren’t”.

Some parents noted difficulty integrating emotion coaching into their existing parenting practices because moving through the five steps felt unnatural, and they were nervous about their child’s reactions to a new parenting approach. Many parents expressed hesitancy in knowing how and when to use emotion coaching. Another parent shared, “So I think there was difficulty … knowing when to apply the method and how my kids will take it. If I didn’t like apply the method”, which may indicate a need to provide live coaching opportunities with parents as they practice implementing the strategies with their children.

#### 3.2.2. Theme 2: Parenting Practices and Beliefs

Throughout the parent interviews, parents shared how the program provided additional strategies to consider in responding to their children’s strong emotions. They also recognized that while they changed their parenting practices, it took time to adapt and become comfortable with the emotion coaching approach. One parent shared that “as time went by, I learned to apply coaching method only to instances that apply and to instances that would not apply, then I was forced to use methods that I know best”. Several parents remarked that adults and parents tend to believe “they know best”, which can lead to misunderstandings between parents and their children. By attending to children’s emotions, parents recognized they were beginning to understand and seek their child’s perspective when emotions arose. One parent said, “Because from a point where you want to understand where the kid is coming from, you also, as a parent, want to … ensure that you get the most out of the relationship”. Additionally, parents noted how the emotion coaching method helps to correct their child and provides an alternate method of helping them problem-solve without parents jumping in immediately to solve the child’s problem as they would have in the past. One parent remarked: “And then so, I think the strategy in the intervention actually goes a long way in correcting some ways and in giving some extra insights on how to deal with problems occurring between parents and children and then the way we like educate them”.

#### 3.2.3. Theme 3: Parental Emotion Regulation

Seventy-eight percent of parents in the TIK group self-reported fewer difficulties with emotion regulation after participating in the program. While the program focuses on teaching children about their emotions and ways to regulate them, time was spent discussing how positive parental emotion regulation is key to effective emotion coaching. Several parents recognized a change in their abilities to regulate their emotions and how it impacts their parenting practices, using many relaxation strategies for themselves and their children. One parent said, “So definitely, when I’m more regulated, and like when I feel like I’m more calm or able to be there for him. I’m definitely more clued in and using tips and tools provided versus just shooting him down”. Similarly, parents recognized a shift in how they interacted with their children when displaying larger emotions. Paired with increased empathy, parents acknowledged a greater intention to listen to their children rather than immediately dismissing their emotions and perspectives. TIK emphasizes taking a moment to pause and regulate before responding to their child, which helps with regulating their emotions. One parent shared how they shifted from tuning out their child to providing more opportunities to listen to their child and prompt for more information: “But I will say there’s been an increase of my listening, like really listening to what he’s saying. And posing questions to him, or telling him, like, it’s okay to feel that way or like, I imagine a lot of other kids are like, I felt that way”.

#### 3.2.4. Theme 4: Child Behavior and Self-Regulation

Parents noted that they recognized an increase and improvement in their children’s ability to express their emotions and the reasons why they felt certain emotions. They noted how this differed from their child’s behavior before the program. One parent noted that their child appeared to regulate their emotions independently after being in this parenting program, and they had a better relationship with their emotions. She noted, “So I think it was okay. If I say, I think it taught my child the skills to kind of bond with their emotions”. Another parent shared how the program has “really been helping me and [my son] with his strong emotions or with his emotions period. I don’t wanna call them strong, but just like with his emotions period”. One parent mentioned, “After this program, I felt that my kid can be able to express themselves confidently in front of others. And again, it says that my kid could- my kid could open up more to me”. On the other hand, some parents indicated that their child’s challenging behavior increased throughout their time in the TIK program, recognizing that more emotional expression may be a brief precursor to increased behavioral challenges. Another parent further stated that their child was more comfortable expressing their emotions, which resulted in more behavioral challenges and stated: “I feel like he just was able to express himself more and tell about his emotions, he felt more comfortable talking about his emotions, and just kind of just feeling more so free to just, I guess, behave in a certain way. Just because he felt comfortable”.

#### 3.2.5. Theme 5: Parent–Child Relationship

Parents expressed that emotions are okay and how their reframing of emotional expression has changed since completing TIK. One parent shared, “It’s okay for my kid to show all the emotion, despite being told, okay, pretend they’re likely not to cry in front of other people. That it’s fine if they show emotion”. Another parent noted how they are “normalizing a bit more of how he’s feeling versus just being like, you’re fine. Or, like, it’s gonna be okay”. Several parents recognized a common belief that shared how they do not believe all people think that children’s strong emotions are unacceptable and how important it is to explore emotions with their child. They shared, “I don’t think a lot of people know or think that having those emotions is okay. And I think that was important and just being okay with talking about their emotions”.

All of the improvements in the parent–child relationship are reflected well in parents’ recognition of their child as a whole person. Several parents reflected on recognizing that they are not all-knowing beings in their relationships with their children. One parent shared insight about how they had to challenge the notion that they are always right, sharing, “I know what is right and what is wrong as a parent, but I realize no, I’m not always right. And that children are people, too. And they should be listened to”. Similarly, another parent shared how children are not always treated like people with emotions in the same way as adults: “And it’s like a kid’s a kid like, people, adults, we have emotions, how can you expect a kid to like not have emotions about things or to not feel things. … Like, they’re people, they’re gonna be the same way. But culturally, we don’t look at, we don’t look at kids as people, like we look at kids as like, I don’t wanna say like robots or like, even possessions in a way. Like, they don’t get to feel things … they’re not allowed to be whole people”.

#### 3.2.6. Theme 6: Acceptability

Because of the virtual nature of the program delivery, the facilitators recorded each session and made the audio recordings available for parents to review after sessions or catch up on material they had missed. One parent expressed how they used the recordings when “the time for the meetings coincided with a part-time job that I had. So I didn’t manage to attend all of them. But I listened to the recordings that were sent”. Because each session built upon the last, parents could keep up with the material by listening to audio recordings and following the slides. Parents appreciated the opportunity to practice the skills in small groups rather than listening to the sessions as lectures. Parents in the program could watch the facilitators role-play scenarios with direct scripts, and they had the opportunity to practice with scripts before moving to real-world examples from their lives to role-play the skills in real situations they have encountered with their children. Another parent shared that using breakout rooms helped separate from the larger group to practice role-playing, and “it was so engaging because in the breakout rooms, you are able to get chances and pick out, rather act it out”. Several parents indicated appreciating the sense of community in a group with other parents experiencing similar challenges with their children. One parent shared that they appreciated hearing from other parents with similar issues and experiences, stating that “the best part was being able to talk to the real people and getting to understand where they come from, about their experiences [with] their family”. It made the breakout rooms more enriching to bounce ideas for implementing the strategies in the real world with their children.

#### 3.2.7. Theme 7: Alignment with Culture and Values

Throughout the program and parent interviews, participants shared how the program aligned with some of their parenting values. In their interviews, parents shared how TIK may differ from how they have done things, but the strategies align with how they have been trying to parent their children. Parents also shared how they have attended parenting classes in the past, and participating in TIK provided them another opportunity to learn about strategies and approaches that align with their values. For example, one parent shared that “we have already been kind of taken steps and … parenting classes. And then when we, you know, kind of saw this program. We were, we were like, Yeah, we could really benefit from it”. Relatedly, some parents spoke about how the program doesn’t necessarily align with their parenting practices, but they see how the framework aligns with their values. One parent shared their perspective using these strategies coming from an older generation with a different way of parenting: “Um, I come from an older generation. So from what I will start from when I was talking about my parents, I wouldn’t say I kind of think that the whole model was based on patience and listening, something that we weren’t like quite used to. But as for me, I would like own them. Yeah. So I think they are aligned with me”.

A few parents talked about how this program allowed them to change their parenting and take a gentle approach. One parent, for example, said, “It’s okay that I can still parent them gently, and they’ll listen to me. Yeah. And I feel that with time, I’m achieving what I wanted instead of having something harsher”. Another parent elaborated on the broader focus for millennial parents to change the landscape of parenting in the future, indicating that “we’re recognizing that the strategies that our parents used may not necessarily have been the most effective … I do feel like our generation is trying to break some of that resistance to emotions”. One parent spoke to the realities and difficulties of raising Black children, specifically Black boys, sharing how Black children are perceived in society and how societal pressures influence parenting approaches. They shared how this program helped them realize how Black males either hide their feelings or dismiss others: ”I thought about this more, especially by coming through this program, and raising Black boys like, then we get upset when we have like males walking around who dismiss other people’s emotions or can’t, put out there, how they’re feeling. And so like, that’s another reason why I’ve been huge on like him expressing himself because I’m like, I’m raising this Black boy who eventually one day will be somebody’s partner”.

## 4. Discussion

Universal evidence-based interventions are those that are not only carried out as intended but also lead to the intended benefits and are found to be acceptable by their participants. This pilot study aimed to examine the fidelity, engagement, efficacy, and acceptability of the Tuning in to Kids parenting program within a group (*n* = 21) of Black, U.S.-based parents of preschoolers. Establishing evidence for using TIK in new contexts and with diverse parent populations is essential to determine its utility across cultures.

Treatment engagement and fidelity are essential when examining an intervention’s efficacy because intervention outcomes should be interpreted cautiously if the intervention is not implemented as planned [67]. If low fidelity is reported with high effectiveness, researchers cannot demonstrate the intervention’s effectiveness in each study [68]. The facilitator delivered TIK virtually with excellent fidelity and across sessions (ratings of 100% or higher), meeting the threshold of high fidelity with ratings of 80% or higher [66]. Further, these fidelity scores are higher than the standard ratings of adequate fidelity and align with previous literature on in-person TIK programs [40,47,69]. Within the TIK literature, most research displays fidelity ratings above 90% within predominantly White samples. Further, these fidelity findings suggest that TIK can be delivered virtually with fidelity using videoconferencing platforms.

In addition to fidelity ratings, 78% (7 out of 9) of parents attended five or more virtual sessions, which is less than the 80% of parents who completed TIK programs in previous literature [43,45,56]. This rate, however, is consistent with, and exceeds, a previous examination of the Incredible Years Parenting program with a majority Black sample, with parents averaging 55% attendance across all sessions [70]. Parents noted that the virtual sessions through Zoom helped accommodate schedules and reduced the need for childcare, transportation, or barriers related to COVID-19 restrictions or concerns [71,72]. Notable differences in outcomes from parents who attended fewer sessions are discussed in the relevant sections below. Delivering parenting groups virtually allowed parents to learn skills and tend to responsibilities without disrupting their schedules. The parent interviews support the importance of providing alternative options for parents who cannot consistently attend in-person parenting groups but still wish to learn the content. However, one parent noted how using a virtual platform makes it difficult to know when other participants are fully engaged. These adequate fidelity scores and attendance rates are promising for virtually delivered parenting programs that are used to reduce implementation barriers often common in group parenting programs [72]. Specifically, this information can support continued examination of treatment adherence and overall attendance for Black parents participating in group-based parenting programs.

While parental emotion regulation plays a key role in parenting practices and children’s emotion regulation it is not well-studied within the parenting intervention literature [31,73,74]. Previous literature suggests that TIK effectively improves parents’ emotion regulation in predominantly White samples [44,56]. The current study’s findings provided preliminary trend data to support TIK’s ability to impact parents’ difficulties with emotion regulation compared to parents in a WLC group. Parents in the TIK group demonstrated overall moderate reductions in difficulties with emotion regulation $\left(\eta_{p}^{2}=0.08\right)$ for Black parents, which adds to the literature addressing the bidirectional relationship between parental and child emotion regulation [73]. Morris and colleagues recognized the impact of parental characteristics such as reactivity and regulation on a child’s emotion regulation and adjustment [12]. Parents in the present study acknowledged the direct impact of their emotion regulation on a change in parenting practices and greater attention to their child’s behavior and emotional processes. Parents’ personal reflections provide preliminary evidence supporting the need to investigate parental emotion regulation in Black parents. Researchers have demonstrated the bidirectional relationship within predominantly homogenous samples; however, the findings of this study align with these previous findings, suggesting that Black parents’ emotion regulation can improve with direct programming (i.e., group intervention or bibliotherapy).

Parents in the treatment group reported a slight increase in parents’ ratings of emotion coaching styles and attitudes and a decrease in emotion dismissing styles and attitudes compared to parents in the WLC group. This finding aligns with previous findings where parents did not report significant changes in emotion coaching [42,43,44] after completing TIK. It also reflects a previous study examining changes in emotion coaching and emotion dismissing in a sample of parents in the U.S. after completing the parent-administered adaptation called TIKOL [75]. Of note, the current study also reflects a greater magnitude of reduced emotion dismissing $\left(\eta_{p}^{2}=0.20\right)$, which is a common finding in the TIK literature with parents reporting greater changes in emotion dismissing than emotion coaching. A possible explanation for the similarities in these studies could be the assessment of skills in a similar sample of parents in the U.S. These results could be due to several possibilities. In the interviews, parents also reported using emotion coaching more and felt themselves dismissing their child less [22,36].Because emotion coaching is generally a practice employed by Black parents [2], these findings suggest that TIK could be an appropriate program to support parents from a strengths-based framework, continuing to build skills that have been shown to be essential to Black parenting.

In addition to these promising results, future investigations can assess whether cultural adaptations could further add to the content from a culturally specific approach. Previous cultural adaptations to the TIK program have included translating material to the language or dialect of the target group, adding content based on culturally specific concepts, and creating videos with caregivers reflecting the intended group [47]. For Black parents in the U.S., adaptations may include using examples that are specific and relevant to the parents in the group, such as using video materials with Black parents, changing language in the videos to reflect African American Vernacular English (AAVE), and incorporating emotion coaching in common situations that Black children and families often face [22]. As several parents discussed the reality of raising Black children, it may be essential to include a session about using emotion coaching in the context of racial socialization, preparing children to respond appropriately in the face of racial discrimination [13] and include more opportunities to role-play illustrating emotion coaching in this context. Parents also shared some difficulty in implementing emotion coaching effectively, which may indicate a need for additional opportunities for role-playing and additional sessions to facilitate a better understanding of using the strategy for children’s stronger emotions [25]. In addition to cultural considerations within the TIK program content and delivery, the group facilitators were both Black Americans to facilitate stronger rapport and build a sense of cultural understanding.

When assessing Black parents’ positive emotion socialization practices, there were positive trends in parents’ reported problem-focused reactions and expressive encouragement from pre- to post-intervention for parents in the TIK group compared to those in the WLC group. There were also reductions observed in negative emotion socialization practices (i.e., distressed emotions, punitive reactions, and minimizing reactions) for Black parents in the TIK group from pre- to post-intervention, which is consistent with other parenting programs in the literature [22,36,75]. The greater magnitude of change demonstrated by parents in the TIK group provides preliminary evidence that TIK can influence Black parents’ emotion socialization practices, especially engaging in emotion-focused reactions $\left(\eta_{p}^{2}=0.17\right)$. Black parents use a myriad of practices to parent their children, and those practices are generally dependent on the situation and the societal implications and potential consequences [4,5,6,7]. Given the need to adjust parenting practices, binary terms such as “negative” and “positive” may not be appropriate in labeling parenting practices as they are often adaptive, especially for Black parents [5]. Future parenting research should consider how using these binary terms can show prejudice toward different parent groups, such as Black parents, who are often positioned as using “negative” parenting practices. To further support TIK’s use with Black parents, future examinations may consider if culturally adapting the program to address using emotion coaching to help navigate systemic barriers may be even more effective for Black parents [2,3].

These positive changes in parents’ reported practices align with other emotion-focused programs [35,70] and other examinations of Black parents’ existing parenting practices, as emotional expression is a pertinent element of African American culture [5]. Parents reported improvements in open communication with their children during the parent interviews. This may be due to the focus on allowing children to sit in the emotions they feel rather than dismissing them. Parents also indicated that their children expressed their emotions more and tried to be more descriptive as they talked about their emotional reactions to situations. Emotion-focused reactions, however, are related directly to the emotion coaching instruction in the program. The increase in reported emotion-focused reactions between post-intervention and the 1-month follow-up may indicate that Black parents need more time to integrate emotion-focused reactions into their parenting styles in addition to problem-focused reactions. Greater changes may be explored in allowing Black parents to practice using the skills with their children in addition to the role-play opportunities in the TIK program.

Because children were not active participants in TIK, it is difficult to determine if behavioral changes resulted from their parent’s involvement in the program or external factors. Additionally, the improved self-regulation for children in both conditions may indicate natural maturation as children get older and are better able to regulate their emotions and behavior. This change may also be due to parents in both groups having access to parenting resources (i.e., TIK and The Emotionally Intelligent Child) throughout the study. According to parent reports in the interviews, several parents indicated that they saw positive improvements in their children’s challenging behaviors and an acknowledgment of their children learning from their parents’ use of emotion coaching strategies. Previous literature demonstrates children’s improvements in emotional competence and emotion labeling when directly involved in parenting programs [35,36,70]. The results of the current study suggest that children do not need to be directly involved in parenting programs to benefit from them. Further investigation should investigate the differences between children whose parents engage with TIK and those of a control group with no additional resources or parenting education.

Like previous TIK findings, parents rated the program as moderately acceptable and were amenable to using the strategies in their parenting despite some misalignment with previous parenting practices. One of the greatest strengths of TIK was the ability to help parents reframe their parenting beliefs and make a shift to healthier and more positive methods of parenting, which is consistent with other qualitative evaluations of parenting programs [22,76]. Specifically, this is like Hernandez and colleagues’ examination of TIK in a rural community in the U.S. Parents in both studies emphasized some initial resistance to the strategies taught but were able to recognize the benefits of reframing their beliefs to adopt emotion coaching within their parenting practices [77]. Like other evaluations of parenting programs, parents expressed how pleased they were with the program and their desire to share the information they learned with family and friends [22]. Lastly, parents were overwhelmingly vocal about the importance of peer support from other parents in the program, even expressing a desire for more peer engagement throughout the program. Creating a positive learning community filled with other parents enhanced their ability to glean pertinent information from the program by acting as peer support and peer models during parenting sessions [21,78]. Black parents can benefit from the virtual delivery of TIK; however, future examinations of the virtual delivery of the program may require participants to leave their cameras on throughout the sessions to foster a more cohesive learning community and better assess individual engagement outside of attendance and parent report. Coupled with positive feedback from parent interviews, Black parents in this sample considered TIK to be highly acceptable for addressing their child’s self-regulation challenges. This data emphasizes how parent engagement and fidelity can contribute to efficacy and acceptability outcomes.

## 5. Study Limitations and Future Directions

While this study provides preliminary support for TIK’s utility for Black parents, it is not without limitations. One limitation of this study is the combination of attrition and a small sample, with 3 out of 21 (14%) parents dropping out after enrolling. Researchers identify time, motivation, and difficulties with employment and transportation as reasons participants may drop out of intervention studies [71], especially during a pandemic [72]. This attrition rate may give insight into difficulties in implementation success in real-world settings for parents despite the virtual approach, which was hoped to promote accessibility. In addition to the challenge of attrition, the small sample (*N* = 21) may have lacked the power to detect significant change for parents or provide sufficient data to make generalizability claims. The inclusion of effect sizes to measure magnitude of change of the TIK intervention is more meaningful that parametric statistics within a small sample. These effect sizes demonstrate promising information for future research to further explore TIK’s utility within larger samples [50]. Future research should engage in effectiveness trials with larger samples in community and clinical settings to determine how likely parents are to drop out of these programs and address barriers to completion, which may further support the promising preliminary data in the current study. In this study, only a 1-month follow-up was used to observe the lasting effects of the TIK outcomes. Given the time restraints of the current study, extending the follow-up period was not possible. Parents’ interviews suggested that increased time to become more comfortable with the material may have resulted in using emotion coaching with more fidelity over time. Future studies should employ longer follow-up periods or booster sessions to ensure parents continued understanding and implementation of emotion coaching beyond the initial six-session program [40].

Additionally, due to the small sample and even smaller sample of parents completing parent interviews, the rigor of qualitative methods was limited to more exploratory procedures. The current pilot sample was generally educated beyond high school and were working full-time, reducing the ability to apply these outcomes to a more socioeconomically diverse group or parents. Future researchers should consider recruiting through community efforts such as churches, community mental health organizations, and local parenting groups for a larger sample. Connecting with community leaders, specifically, may aid in garnering buy-in from valued community members. With a larger sample of interviews specifically, future examinations should consider employing a more rigorous qualitative methodology, such as data-checking with participants, incorporating an iterative process in data planning and analyses, and engaging in a triangulation framework to better establish validity of interpretation between quantitative and qualitative data. Another limitation is the limited data collection measures. Future research should consider using observational data and multiple reporters to measure parents’ use of emotion coaching and children’s behavior. Using multiple methods to measure parent and child outcomes will strengthen data collection and analyses in future research and help to combat potential social desirability bias. While technology served as an asset for parents participating in the study, there were notable challenges with recruiting participants through email contacts, social media, and other media-related recruitment efforts.

## 6. Study Implications

The results of this study build upon the vast research examining the effectiveness of TIK and other emotion-focused parenting programs. This is the first study to evaluate TIK with a Black U.S.-based sample. Because of the small sample size, a more appropriate way to report non-significant results is to report the observed differences with effect sizes and then carefully highlight which results were predicted to be different. Given the medium to large effect sizes within the measured outcome data, this pilot study provides preliminary evidence of its utility in a sample of Black parents in the United States. As parenting literature grows, it is critical to examine parenting programs and their utility for parents from diverse backgrounds. The literature on emotion-focused parenting programs is conducted with primarily White samples; thus, our study provides promising data to support further investigation of TIK’s utility in a new population. Because emotion socialization exists within the cultural context of a child’s family or community, parenting programs for Black parents should. Address poignant difficulties faced in a sociocultural context [22]. The prospect of investigating TIK incorporating cultural adaptations specific to Black parents in the U.S. may lead to a better understanding of concepts when presented in culturally relevant scenarios and paired with program materials, including Black actors in videos and AAVE. Additionally, findings contribute to the growing body of literature supporting the virtual delivery of parenting programs. Given the technological age and future advancements, virtually delivered programs provide a gateway to accessing effective programs for parents and caregivers to help them best support their children. Our study indicated that manualized parenting programs can be delivered via teleconferencing with excellent fidelity.

Many parenting programs focus on behavior management, and TIK provides an alternative pathway for supporting parents while engaging in psychoeducation about children’s social and emotional development. Clinicians may consider facilitating these groups in community or clinical settings to help parents and caregivers better understand their children’s emotional development when behavior is not the primary concern for a child. The qualitative data collected in this study sets the stage for clinical implications related to adapting TIK to best fit parents’ needs. One consideration for using TIK is to use the flexible nature of the manual to adapt the content to fit parents’ needs. Generally, parents reported that TIK was sensitive to their cultural beliefs; however, future examinations should consider creating and using visual materials (e.g., handouts, video scenarios) with parents from diverse backgrounds to help make the information more relatable for parents [76].

## 7. Conclusions

We used a mixed-method, randomized-waitlist pilot trial to assess the fidelity, engagement, efficacy, and acceptability of the Tuning in to Kids parenting program for Black parents of preschoolers in the U.S. The results of this study indicated that TIK had high facilitator fidelity, parent-reported engagement, and adequate attendance. Predominantly medium to large effect sizes in improvement of parental emotion regulation, use of emotion coaching principles and positive parenting practices, and children’s emotional competence and self-regulation were found in the TIK group. The changes demonstrated in this pilot study combined with positive perceptions of TIK’s acceptability provide further evidence for TIK’s intended positive impact on Black parents’ emotion socialization practices and beliefs. These findings add to the TIK literature by assessing the program’s impact in a new sample of Black parents in the United States, demonstrating similar findings for parents and children in prior literature. This study also adds to the literature examining the effectiveness and utility of virtually delivered parenting programs by providing further evidence supporting their utility and reducing access barriers.

## Figures and Tables

**Table 1:** Demographics and Characteristics for Participants in the TIK and WLC Groups.

	TIK (*n* = 9)		WLC (*n* = 12)	
	M	SD	M	SD
Parent				
Age (years)	32.4	6.2	32	5.2
Female: *n* (%)	5 (55.6)		5 (41.7)	
Male: *n* (%)	4 (44.4)		7 (58.3)	
Child				
Age (months)	48.2	8.1	39.9	5.6
Female: *n* (%)	2 (22.2)		4 (33.3)	
Male: *n* (%)	7 (77.8)		8 (66.7)	
Relationship to child: *n* (%)				
Biological Mother	5 (55.6)		5 (41.7)	
Biological Father	4 (44.4)		7 (58.3)	
Ethnicity				
African American	9 (100%)		11 (91.7)	
Jamaican	-		1 (8.3)	
Education: *n* (%)				
HS or GED	1 (11.1)		-	
Some College	-		1 (8.3)	
College	5 (55.6)		7 (58.3)	
Graduate School	3 (33.3)		4 (33.3)	
Employment: *n* (%)				
Full-time	8 (88.9)		9 (75)	
Part-time	1 (11.1)		3 (25)	

**Table 2:** Attendance and Percentages of Group Facilitator Fidelity Scores for the TIK Group.

Session (Title)	*n*	Fidelity
Session 1 (Setting Out: How to Raise Emotionally Intelligent Children)	6	100%
Session 2 (Naming the Emotion)	9	100%
Session 3 (Understanding Your Child’s Emotional Experience)	8	100%
Session 4 (Self-care, Problem-Solving, and Coaching Fears and Worries)	6	100%
Session 5 (Emotion Coaching and Your Child’s Anger)	8	100%
Session 6 (Emotionally Intelligent Parenting: Now and in the Future)	8	100%
Total		100%

**Table 3:** Means and standard deviations of parent and child outcomes with effect sizes.

	TIK Group (*n* = 9)			WLC Group (*n* = 12)			
	Pre	Post	F-Up	Pre	Post	F-Up	Effect Size
Variable	M (SD)	M (SD)	M (SD)	M (SD)	M (SD)	M (SD)	$\eta_{p}^{2}$
Difficulties in Emotion Regulation Scale							
NER	14.6 (4.2)	13.8 (5.2)	12.4 (3.4)	13.8 (5.9)	14.1 (5.2)	13.8 (5.3)	0.10 ^a^
GDB	14.3 (6.1)	15.2 (3.7)	11.4 (2.7)	12.5 (3.4)	13.6 (2.8)	12.6 (3)	0.14 ^b^
ICD	14 (4.4)	12.6 (4.6)	11 (4.6)	14.5 (4.9)	14.2 (4.9)	12.9 (5.7)	0.03 ^a^
LEA	14 (3.4)	13.7 (3.1)	12 (3.5)	15.2 (4.0)	14.1 (3.1)	14.1 (4.6)	0.08 ^a^
LAERS	20.8 (8.1)	17.1 (5.2)	16.6 (4.3)	18.3 (6.0)	17.3 (6.3)	17.9 (6.1)	0.09 ^a^
LEC	11.2 (3.7)	9.4 (3.4)	10.9 (4.5)	11.8 (3.4)	10.8 (3.4)	11 (3.7)	0.05 ^a^
Total	88.9 (23.4)	81.8 (19.6)	74.3 (18.6)	86.2 (21.4)	84 (19.7)	82.3 (23.8)	0.08 ^a^
Parental Emotional Style Questionnaire							
EC	44.6 (4.4)	45.2 (5.9)	45.0 (5.7)	45.0 (6)	45.0 (6.1)	44.5 (8.8)	0.01
ED	41 (5.0)	35.9 (5.5)	37.1 (5.8)	38.8 (7.6)	38.3 (6.0)	39.9 (7.6)	0.20 ^b^
Coping with Children’s Negative Emotions Scale							
EFR	5.6 (0.7)	5.0 (0.6)	5.4 (0.7)	5.3 (1.4)	5.4 (1.2)	5.4 (1.2)	0.17 ^b^
PFR	5.5 (0.6)	5.7 (0.6)	5.7 (0.9)	5.2 (1.2)	5.2 (1.1)	5.3 (1.2)	0.02
EE	4.9 (1.1)	5.5 (0.9)	5.3 (1.0)	4.5 (0.9)	4.9 (0.9)	4.9 (1.0)	0.07 ^a^
DR	4.0 (0.8)	3.2 (0.9)	3.2 (0.9)	3.2 (1.1)	3.2 (1.0)	3.0 (0.9)	0.13 ^a^
PR	3.3 (1.2)	2.7 (1.4)	2.9 (1.1)	3.7 (1.6)	3.6 (2.0)	3.7 (1.9)	0.06 ^a^
MR	3.1 (1.1)	2.9 (1.5)	3.2 (1.2)	3.8 (1.5)	3.8 (1.7)	3.8 (1.7)	0.04
Devereux Early Childhood Assessment for Preschoolers, Second Edition							
SR	35 (3.2)	54 (2.8)	56 (5.0)	36 (4.7)	62 (3.7)	64 (3.9)	0.21 ^b^
BC	62 (4.8)	70 (5.1)	71 (4.3)	63 (5.5)	72 (6.4)	72 (6.4)	0.02
Attach	28 (4.5)	51 (6.2)	62 (5.9)	28 (4.3)	51 (3.7)	54 (4.2)	0.03
Initiative	34 (4.7)	57 (3.7)	57 (5.1)	34 (4.5)	57 (2.9)	57 (3.8)	0.04
TPF	28 (10.7)	28 (11.5)	29 (12.0)	28 (12.6)	29 (8.0)	30 (8.6)	0.02

*Note.* NER = nonacceptance of emotional responses. GDB = difficulty engaging in goal-directed behavior. ICD = impulse control difficulties. LEA = lack of emotional awareness. LAERS = limited access to emotion regulation strategies. LEC = lack of emotional clarity. EC = emotion coaching. ED = emotion dismissing. EFR = emotion-focused reactions. PFR = problem-focused reactions. EE = expressive encouragement. DR = distressed reactions. PR = punitive reactions. MR = minimization reactions. TPF = Total Protective Factors. SR = Self-Regulation. BC = Behavioral Concerns. Attach = Attachment/Relationships. Effect sizes are considered small (0.01–0.06), moderate (>0.06–0.14), and large (>0.14). ^a^ Moderate effect size. ^b^ Large effect size.

**Table 4:** Themes and Associated Codes.

Theme	Code
Intervention engagement and barriers	Engaging in facilitated discussions based on shared experience Perceptions of others’ lack of participation/engagement Need for more support with continued challenging behavior Challenges integrating emotion coaching
Parenting practices and beliefs	Increased use of emotion coaching Greater attention to child’s emotions Understanding the child’s perspective Recognition that emotions are okay Reduction of dismissive practices
Parental emotion regulation	Pausing as a facilitator of emotion regulation Recognition of own regulation’s impact Use of relaxation strategies
Child behavior and regulation	Improved emotion expression Improved emotion regulation Improvements in child’s behavior Continued behavioral challenges
Parent–child relationship	Improved communication with child Increased empathy toward child Listening to child more Viewing the child as a person
Acceptability	Increased accessibility due to virtual delivery Sense of community and peer support with other parents during breakout rooms Ability to have specific strategies to use to address child’s emotions each session
Alignment with culture and values	Alignment with parenting values Reflecting parenting want for the future Misalignment with community parenting values Realities of raising Black children Approach differing from parents’ upbringing

## Data Availability

Please reach out to the first author about data availability. The data are not publicly available due to privacy and participant confidentiality.
